# Roussy-Lévy Syndrome: Pes Cavus, Tendon Areflexia, Amyotrophy, Gait Ataxia, and Upper Limb Tremor in a Patient with CMT Neuropathy

**DOI:** 10.5334/tohm.846

**Published:** 2024-02-08

**Authors:** Rohini Kumar, Jamie Blackband, Varun Jain, Lee Kugelmann, Sub H. Subramony, Aparna Wagle Shukla

**Affiliations:** 1 Department of Neurology, Norman Fixel Institute for Neurological Diseases, University of Florida, Gainesville, Florida, United States of America

**Keywords:** neuropathy, tremor, CMT, Charcot-Marie-Tooth disease, Roussy-Lévy syndrome

## Abstract

**Background:**

Roussy-Lévy syndrome (RLS) is characterized by postural hand tremor seen in patients with familial autosomal dominant Charcot-Marie-Tooth (CMT) neuropathy.

**Phenomenology Shown:**

This video demonstrates irregular, jerky bilateral kinetic, postural, rest tremor affecting the right > left hand, along with pes cavus and gait ataxia in a patient with CMT disease.

**Educational Value:**

Pes cavus, tendon areflexia, sensory ataxia, and upper limb tremor should prompt consideration of CMT neuropathy.

**Highlights:**

This video abstract depicts a bilateral hand tremor characteristic of Roussy-Lévy syndrome seen in patients with Charcot-Marie-Tooth disease neuropathy. The significance of the abstract lies in the phenomenology and the physiology of the tremor seen in patients with genetically confirmed duplication of PMP22 gene.

Roussy-Lévy syndrome was first described in 1926 in seven members of a large kindred with dominantly inherited neuropathy. These family members presented with prominent features of an unsteady gait manifesting during childhood, alongside pes cavus, generalized areflexia, distal amyotrophy and weakness, clumsiness, and postural tremor with limb ataxia. The familial neuropathy is related to segmental duplication of the PMP22 gene in 70% of cases (CMT1A). PMP22 duplications, accounts for the most common inherited demyelinating neuropathy in North America. In other cases, a point mutation of the PMP22 or P0 gene results in neuropathy [1,2]

A 25-year-old male with a diagnosis of Charcot Marie Tooth disease (CMT; attributed to the PMP22 gene) presented with distal progressive numbness, weakness, and muscle wasting in the legs beginning when he was 11 years old. He soon began to experience balance difficulties and pain in the feet that ascended to his knees. By the age of 16, he noticed weakness in his hands and difficulty gripping objects. At age 18, he began to notice hand tremors, primarily action tremors, that led to difficulty opening jars, handling utensils, writing, and typing. The patient’s mother and grandmother had similar neuropathy symptoms that spanned several decades. Physical examination revealed a bilateral kinetic, postural, and rest tremor affecting the right > left hand. The tremor was irregular, jerky, and asymmetric. Tremor severity was rated with the Fahn-Tolosa-Marin scale, where scoring was 2 for kinetic, 2 for postural, and 1 for rest tremor. Further clinical examination revealed features of CMT disease, such as pes cavus, tendon areflexia, slight amyotrophy, and gait ataxia, which was primarily sensory and not cerebellar. Gait was wide-based, but the tandem task was somewhat possible (Video 1). A nerve conduction study revealed a demyelinating neuropathy affecting the peroneal, median, and ulnar nerves. The nerve conduction velocity was < 38 m/sec, a finding characteristic of CMT neuropathy. A power spectrum analysis of postural tremor accelerometer recordings revealed a peak around 5 Hz (Figure 1).

**Video 1:** **Tremor in Roussy-Lévy syndrome**. Tremor is shown at rest in the right hand; however, it is more proximal than typically seen in Parkinson’s disease. With arms outstretched, there is a mild to moderate postural tremor on the right. Unlike essential tremor, it has a jerky quality and the predominant movement is wrist pronation-supination rather than flexion-extension. In the wingbeat position, a jerky tremor of mild to moderate amplitude is seen on the right. Mild kinetic tremor (right greater than left) is seen on the finger-nose maneuver; the tremor is jerky and there is no intentional component. During spiral drawing, moderate amplitude tremor is seen on the right and mild tremor on the left. During dot approximation, mild tremor is seen on the right and trace on the left. Gait is wide-based, and the patient was unable to tandem walk without needing to touch the wall with his left hand.

**Figure 1:**
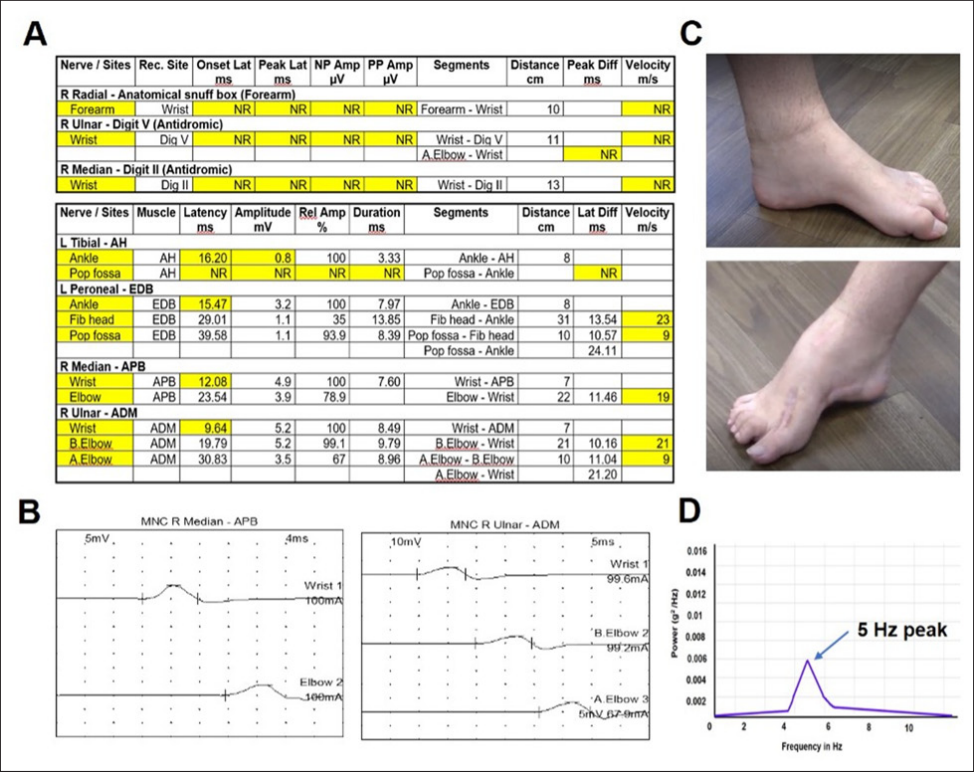
A: Nerve conduction study consistent with primarily demyelinating neuropathy. Nerve conduction velocity in peroneal, median and ulnar nerves < 38 m/sec; characteristic of CMT1A neuropathy.

These findings of tremor manifesting in the setting of familial neuropathy are consistent with a diagnosis of Roussy-Lévy syndrome [1]. A bilateral action tremor affecting the hands, even though emerging in the setting of a familial neuropathy, could be potentially mislabeled as an essential tremor. However, some clinical features were not typical of ET, including the jerky nature of the tremor, pronation-supination of the wrist rather than flexion-extension, and severity of rest tremor relative to kinetic tremor. Our patient’s peak frequency of 5 Hz is on the lower side compared to a broader range of 4–12 Hz reported in essential tremor [2]. Whether a CMT tremor is more like a dystonic tremor than essential tremor is a question that can be sorted by conducting larger studies.

The mechanisms underlying tremor seen in familial neuropathy need investigation. While our patient did not have prominent cerebellar features of gait ataxia or limb dysmetria, as applicable to tremors arising in the setting of inflammatory neuropathies [3], cerebellar dysfunction is a leading hypothesis. As such, the cerebello-thalamo-cortical pathway is considered one of the main central pathways for the generation of many tremor disorders, including essential tremor and Parkinson’s disease tremors. Currently there are no definite treatments for addressing the tremor. Nevertheless, our patient was provided a trial of propranolol 20 mg twice daily, which led to mild clinical improvement.
